# A Systems Approach Implicates a Brain Mitochondrial Oxidative Homeostasis Co-expression Network in Genetic Vulnerability to Alcohol Withdrawal

**DOI:** 10.3389/fgene.2016.00218

**Published:** 2017-01-03

**Authors:** Nicole A. R. Walter, DeAunne L. Denmark, Laura B. Kozell, Kari J. Buck

**Affiliations:** ^1^Research and Development, Portland Veterans Affairs Medical Center, PortlandOR, USA; ^2^Department of Behavioral Neuroscience, School of Medicine, Oregon Health and Science University, PortlandOR, USA

**Keywords:** alcoholism, withdrawal, *N*-acetylcysteine, oxidative stress, congenic, mouse models

## Abstract

Genetic factors significantly affect vulnerability to alcohol dependence (alcoholism). We previously identified quantitative trait loci on distal mouse chromosome 1 with large effects on predisposition to alcohol physiological dependence and associated withdrawal following both chronic and acute alcohol exposure in mice (*Alcdp1 and Alcw1*, respectively). We fine-mapped these loci to a 1.1–1.7 Mb interval syntenic with human 1q23.2-23.3. *Alcw1/Alcdp1* interval genes show remarkable genetic variation among mice derived from the C57BL/6J and DBA/2J strains, the two most widely studied genetic animal models for alcohol-related traits. Here, we report the creation of a novel recombinant *Alcw1/Alcdp1* congenic model (R2) in which the *Alcw1/Alcdp1* interval from a donor C57BL/6J strain is introgressed onto a uniform, inbred DBA/2J genetic background. As expected, R2 mice demonstrate significantly less severe alcohol withdrawal compared to wild-type littermates. Additionally, comparing R2 and background strain animals, as well as reciprocal congenic (R8) and appropriate background strain animals, we assessed *Alcw1/Alcdp1* dependent brain gene expression using microarray and quantitative PCR analyses. To our knowledge this includes the first Weighted Gene Co-expression Network Analysis using reciprocal congenic models. Importantly, this allows detection of co-expression patterns limited to one or common to both genetic backgrounds with high or low predisposition to alcohol withdrawal severity. The gene expression patterns (modules) in common contain genes related to oxidative phosphorylation, building upon human and animal model studies that implicate involvement of oxidative phosphorylation in alcohol use disorders (AUDs). Finally, we demonstrate that administration of N-acetylcysteine, an FDA-approved antioxidant, significantly reduces symptoms of alcohol withdrawal (convulsions) in mice, thus validating a phenotypic role for this network. Taken together, these studies support the importance of mitochondrial oxidative homeostasis in alcohol withdrawal and identify this network as a valuable therapeutic target in human AUDs.

## Introduction

Alcohol use disorders (AUDs) are a leading cause of global disease burden, disability-adjusted life years, and years lost to disability (Whiteford et al., 2013). Currently, three medications (naltrexone, acamprosate, and disulfiram) are approved by the FDA for the treatment of alcohol dependence, and these primarily target reduction of heavy drinking days. Additional investigational drugs may reduce relapse after a period of abstinence (e.g., ondansetron, baclofen, and topiramate; Johnson et al., 2003, 2007; Addolorato and Leggio, 2010; Batki and Pennington, 2014; Soyka and Lieb, 2015). Unfortunately, these medications have little or no therapeutic efficacy in some patients and/or cause serious side effects, nor do they directly target the alcohol withdrawal symptoms associated with physiological dependence.

Alcoholism is a heterogeneous disorder with a complicated relationship between biological (e.g., genetic) and environmental factors. Human studies have generally identified markers associated with AUD diagnoses and endophenotypes. Social environment, stress, mental health status, and age are all known to impact development and severity of AUDs. Based on family and twin studies, about 50% of AUD risk is genetically influenced (Agrawal and Lynskey, 2008; Verhulst et al., 2015). Despite this high heritability, the genetic determinants of AUD risk remain largely unknown, hindering the development of effective therapeutic and preventative strategies (Ducci and Goldman, 2012).

Because human studies are often retrospective, underpowered, and confounded by comorbid disorders (e.g., abuse of additional substances), animal models are invaluable tools toward the discovery and validation of risk genes and pathways. Seizures, a well-known consequence of dependence and one of the most feared manifestations of withdrawal in alcoholics, are considered “rebound” phenomena owing to ethanol-induced compensatory (homeostatic) processes in the brain. Physiological and behavioral withdrawal signs in mice are similar to those in humans, and many species exhibit convulsions during severe withdrawal and reduced seizure thresholds even during mild withdrawal (Heilig et al., 2010). Although no animal model duplicates clinically defined alcoholism, behavioral assessment of specific factors thought to constitute the motivational forces that perpetuate use and contribute to relapse are useful surrogates for identifying potential genetic determinants of liability in humans. Animal models for alcohol withdrawal symptoms are also useful to determine the effectiveness of potential pharmacotherapies designed to target these forces. Due to their striking divergence in a number of alcohol-related traits, including predisposition to physiological dependence and associated withdrawal, the DBA2/J (D2) and C57BL/6 (B6) mouse strains are the two most widely utilized genetic animal models of AUDs.

Using B6D2-derived genetic models, we previously detected and localized quantitative trait loci (QTLs) on mouse chromosome 1 that significantly affect withdrawal severity following both chronic (*Alcdp1*; Buck et al., 2002) and acute (*Alcw1*; Buck et al., 1997) alcohol exposure. Subsequently, using a panel of novel chromosome 1 QTL interval-specific congenic strains, we finely mapped *Alcdp1* and *Alcw1* to the same 1.1 Mb interval (Kozell et al., 2008). The fact that the QTLs map to the same interval, herein referred to as *Alcw1/Alcdp1*, is consistent with the idea that the same gene or genes contribute to the mechanism(s) of action and phenotypic effects of both QTLs. The present studies are the first to use small donor segment congenics to isolate QTLs on *reciprocal* genetic backgrounds, thus utilizing two *Alcw1/Alcdp1* models, one of which (R8) possesses the smallest (1.1–1.7 Mb) recombinant congenic interval on a B6 background (Kozell et al., 2008). For the second *Alcw1/Alcdp1* model, we report the creation of the first reciprocal congenic (R2) on a D2 background. Due to the near-elimination of confounding genetic background effects, congenic models are extremely powerful tools for elucidating the gene or genes underlying QTL phenotypic effects (Shirley et al., 2004; Kozell et al., 2008, 2009; Doyle et al., 2014; Kato et al., 2014; Kobayashi et al., 2014). QTLs affecting a variety of phenotypes and behaviors in addition to *Alcw1/Alcdp1* have been localized to distal mouse chromosome 1 (Mozhui et al., 2008), making this an attractive target for investigation. Numerous studies have also found significant associations with AUD risk across a broad area of human 1q (Ehlers et al., 2010).

Quantitative trait loci mapping has become a common approach to identify chromosomal regions with a gene(s) influencing a complex trait such as AUD (Milner and Buck, 2010). Identification of quantitative trait genes (QTG) can provide valuable genetic targets for therapeutic interventions. However, often the effects of a single QTG may not be strong enough to detect or strong enough to disrupt the phenotype. However, complementary system genetics approaches such as network analyses can detect important, more subtle gene expression changes to identify biological mechanisms affecting the phenotype and introduce new potential targets for disruption. Similar to QTL analyses, systems genetics integrates genomic and phenotypic data to analyze complex traits (Nadeau and Dudley, 2011; Civelek and Lusis, 2014). For the microarray data presented here, we used weighted gene co-expression network analysis (WGCNA), a systems biology method to describe correlations beyond differential expression (DE) (Langfelder and Horvath, 2008). WGCNA identifies subtle patterns of gene expression clusters (modules), which change coherently and are directly impacted by genotype. We then assessed these modules for biological function to identify mechanisms or pathways contributing to alcohol withdrawal vulnerability.

Molecular network analyses are an important complement to standard QTG identification in translational approaches to complex disease (Emilsson et al., 2008). In some cases, a QTG may be the same in mouse and human (Mogil et al., 2003), while in others, determining additional players and dynamics of the larger network in which candidate QTGs operate may provide more relevant translational utility (Sieberts and Schadt, 2007). Thus, integrating evidence for the influence of an individual gene located within the QTL with that of the co-expression network of the gene can improve understanding of the mechanism by which that gene affects complex traits. The present studies seek to elucidate a mechanism(s) involved in the actions of a QTL with a large effect on genetic predisposition to alcohol withdrawal.

## Materials and Methods

### Animals

B6 and D2 strain breeders were purchased from the Jackson Laboratory. The R2 congenic and the previously developed reciprocal R8 congenic strain (Kozell et al., 2008) were created in our colony at the Veterinary Medical Unit of the Portland VA Medical Center. All congenic and wildtype (WT) animals used were bred in our colony or purchased from Jackson Laboratory. The R8 congenic contains a 1.1 Mb of D2 chromosome 1 introgressed onto an inbred B6 background (minimal 170.9–172.13 Mb; maximal 170.4–172.14 Mb; build GRCm38) (Kozell et al., 2008). Here, we used our D2.B6^-^*^D1Mit206^* congenic strain (Kozell et al., 2008) as the point of departure to develop the reciprocal congenic (R2). Individual animals with recombination within the starting congenic interval were backcrossed to produce multiple offspring with the same recombinant genotype.

R2 heterozygotes were intercrossed to generate the R2 homozygotes, R2 heterozygotes, and WT littermates used for behavioral phenotypic comparisons. Once the congenic strain was established, they were inbred for no more than three generations before being backcrossed to background strain. Heterozygous offspring were intercrossed, and their full-congenic offspring were used as breeders for mice used in the molecular studies. Congenic and background strain mice were housed in the same room. Background strain animals were inbred for three generations. This breeding scheme is much more conservative than that recommended by Jackson Laboratories (10 generations) to limit genetic drift.

Only male mice were used in molecular analyses. Both males and females were used for behavioral studies. Mice were group-housed 2–4 per cage by sex. Mouse chow (Purina #5001) and water were available *ad libitum*, and lights were on from 6:00 to 18:00 with the room temperature maintained at 22.0 ± 1.0°C. All procedures were approved by the VA Medical Center and Oregon Health & Science University Institutional Animal Care and Use Committees in accordance with United States Department of Agriculture and United States Public Health Service guidelines.

### Baseline and Alcohol Withdrawal Enhanced Convulsions

Physiological dependence is defined operationally as the manifestation of physical disturbances (withdrawal) after alcohol administration is suspended. Genetic variation in alcohol withdrawal severity was examined by monitoring handling-induced convulsions (HICs) associated with withdrawal, which is a sensitive index of withdrawal severity (Goldstein and Pal, 1971; Crabbe et al., 1991a). McQuarrie and Fengl (1958) first reported that alcohol withdrawal is apparent in mice following a single hypnotic dose, and this was later shown to be genetically determined (Crabbe et al., 1991b). The initial detection and fine-mapping of a QTL on distal chromosome 1 affecting alcohol withdrawal utilized this acute model (Buck et al., 1997; Kozell et al., 2008). This QTL also affects predisposition to withdrawal following chronic alcohol exposure (Buck et al., 2002; Kozell et al., 2008). Importantly, to avoid confounding by tolerance observed in chronic models (Crabbe et al., 1991a), and to assess central nervous system (CNS) alcohol-sensitivity, the present studies utilize the acute model. Details of this acute alcohol withdrawal procedure have been published (Kruse et al., 2014). In the R2 congenic comparison, R2 homozygotes, heterozygotes, and WT littermates were tested. The mice were scored twice for baseline (pre-ethanol) HICs 20 min apart, followed by a single sedative-hypnotic dose of ethanol (4 g/kg, i.p., 20% v/v in saline) and scored hourly between 2 and 12 h post-ethanol administration. In order to create an index of alcohol withdrawal for each animal that is independent of potential individual differences in baseline HIC scores and reflects differences in withdrawal convulsion severity, post-ethanol HIC scores were corrected for the individual’s average pre-ethanol (baseline) HIC score as in previous work (Kruse et al., 2014). Individual alcohol withdrawal severity scores were then calculated as the area under the curve (i.e., the sum of the post-ethanol HIC scores) from 1 to 12 h post-ethanol.

Additionally, we tested the potential influence of *N*-acetylcysteine (NAC) on baseline and alcohol withdrawal enhanced HICs. The NAC dose and number and timing of NAC administrations were selected based on previous work demonstrating a significant increase in endogenous glutathione in rodent brain (Pocernich et al., 2000) and on our pilot empirical results. Male and female D2 strain mice were used to facilitate detection of either a decrease or increase in withdrawal severity. Using a 2 × 2 factorial design, approximately half of the mice were administered NAC (300 mg/kg, i.p., at both -48 and -24 h relative to ethanol administration) and the other half received vehicle (10 ml/kg saline at both -48 and -24 h). Baseline HIC scores and alcohol withdrawal severity scores were calculated as described above, except a subset of mice was also scored at 24 h to verify that HICs returned to baseline.

### Behavioral Data Analyses

Withdrawal severity scores were determined to be normally distributed based upon a non-significant Chi-Square test and analyzed using an Analysis of Variance (ANOVA) (Systat 13, Systat Software Inc.). When significance was indicated (*p* < 0.05), this was followed by *post hoc* (Tukey) analysis, with significance set to *p* < 0.05 (two-tailed).

### Genotypic Analyses

DNA for genotyping was extracted from ear punch tissue using the Epicentre Quick Extract protocol according to the manufacturer’s instructions. Genotype analyses were performed using standard protocols for simple sequence length polymorphisms (e.g., MIT markers) and single nucleotide polymorphisms (SNPs) using fluorescent probes (Applied Biosystems Taqman methods).

### Gene Expression Microarray and Quantitative PCR (QPCR) Analyses

A total of 32 naïve male animals were used for Illumina Mouse Ref8 v2 array analyses (10 R8, 10 B6, 6 R2, and 6 D2). A separate group of 22 naïve males was used (*n* = 11 per strain) for confirmation testing using quantitative PCR (QPCR) and additional complementary analyses. Whole brain was isolated immediately after cervical dislocation and frozen in liquid nitrogen, and total RNA isolated from individual animals using a standard Trizol method as in previous work (Daniels and Buck, 2002). cRNA was hybridized to Illumina Mouse Ref8 v2 arrays by the OHSU Microarray Core exactly per manufacturer instructions. Data preprocessing steps closely followed that used in previous work (Iancu et al., 2010). Using the R application environment^1^ and Bioconductor^2^, outlier samples were removed and samples normalized using lumi (Du et al., 2008). For genotype dependent DE on the microarray data, we chose an uncorrected *p* = 0.01 threshold based on previous detection of validated DE comparing R8 and background strain animals with a similar threshold (Denmark and Buck, 2008) and on the minimal genetic variation (<1%) between congenic and WT strains. QPCR was used for validation testing of putative DE genes, as well as to query additional genes of interest not represented on the array, as in previous work (Denmark and Buck, 2008).

### Weighted Gene Co-expression Network Analysis (WGCNA)

Parallel WGCNA (Zhang and Horvath, 2005; Langfelder and Horvath, 2008) were performed similarly to previous work (Iancu et al., 2012; Metten et al., 2014) using the R8B6 and R2D2 datasets. Expression data were first filtered for detection across all samples. Gene (probe) expression variability is described by the coefficient of variation (CV), and this variation is lower among congenic and background strain animals than for more genetically diverse populations (e.g., the HSNPT 8-strain cross; Iancu et al., 2013). Therefore, to optimize network robustness (Fuller et al., 2007) and consistency with variation thresholds (Iancu et al., 2013), only probes with a CV ≥ 0.013 were considered adequately variable and included in the network analyses. Pearson correlations were computed between all gene pairs and subsequently raised to a power beta (β = 10) chosen in accordance with the scale free criteria (Zhang and Horvath, 2005), resulting in an adjacency matrix. To detect modules, this matrix was clustered utilizing the hybrid adaptive tree cut procedure following Langfelder and Horvath (2008), with clustering parameters: minimum module size = 20, deepSplit = 4, cutHeight = 0.9999. Each module was arbitrarily assigned a color, and expression properties condensed into a representative profile or module eigengene (ME), reflecting the first principal component of each module (Langfelder and Horvath, 2007). Subsequently, modules with a correlation greater than 0.80 (cutHeight = 0.20) were merged.

### Module Quality and Overlap

To validate network construction, we assessed module robustness in the consensus networks for B6 and R8 (R8B6) and D2 and R2 (R2D2). In each case, gene co-expression modules were compared to random groups of genes from the respective network analyses (Langfelder et al., 2011). Module quality was quantified as *Z* scores, which were derived by comparing module properties (e.g., connectivity) with the same properties derived from 300 sets of random genes approximately the same size as the true modules. Shared membership between R8B6 and R2D2 modules was assessed for each module pair and significance calculated using Fisher’s exact test to assign a p-value to each pairwise overlap.

### kME, kWithin, Gene Significance

Details of our approach (based upon Langfelder and Horvath, 2008; Langfelder et al., 2011) are provided in previous work (Metten et al., 2014). In these, module membership (kME) is a natural measure of connectivity that describes how closely a probe/gene resembles the ME, and intramodular connectivity (kWithin) is calculated as the sum of connection strengths within distinct modules (Langfelder and Horvath, 2008). These measures tend to be highly correlated in co-expression networks, allowing identification of the most highly connected genes, i.e., hubs, likely to have functional and biologically relevant systems-level influence. Gene significance *p*-values (GSP) were generated for each probe in the network analyses (Langfelder and Horvath, 2008), indicating the level of significance of the correlation of a probe to a related trait (genotype).

### Module Preservation

In order to evaluate whether modules in the R8B6 co-expression network were preserved in the R2D2 co-expression network, module preservation statistics were calculated in WGCNA (Langfelder et al., 2011). Z summary (an aggregation of multiple preservation *Z* statistics) was calculated based on 100 permutations using adjacency data. The higher the value of a *Z* statistic, the stronger the evidence that the observed value of the preservation statistic is significantly higher than expected by chance.

### Functional Assessment of Modules

We used Gene Weaver (Baker et al., 2012) to identify curated, published datasets similar to our modules based on gene membership. Beyond these, we also assessed potential mechanisms of co-expression driven by *Alcw1/Alcdp1* genes and that in other published datasets by identifying the intersection of each module with 50 Hallmark gene sets in the Molecular Signatures Database (MSigDB) (Subramanian et al., 2005). Hallmark gene sets summarize and represent specific, well-defined biological states or processes and are generated by a computational methodology that identifies gene set matches. Lastly, we quantitatively assessed cell type enrichment of module members using gene markers for mouse neuronal cell types (Cahoy et al., 2008) and Fisher’s exact test followed by Bonferroni correction.

## Results

### Reciprocal Congenics (R2 and R8) Capture an Alcohol Withdrawal QTL

**FIGURE 1:**
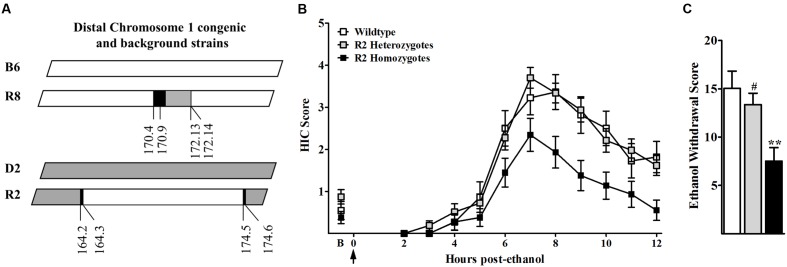
**(A)** R8 congenic animals possess a small 1.1 Mb donor (D2, gray) segment from distal chromosome 1 on an inbred B6 (white) genetic background. The reciprocal congenic, R2, possesses a larger 10.2 Mb donor (B6, white) interval from the same region of distal chromosome 1 on an inbred D2 (gray) genetic background. Boundary regions (within which the recombination site is located) are indicated in black. **(B)** Mice were scored twice for baseline HICs (indicated by B) immediately before administration of 4 g/kg ethanol (arrow = injection at time 0). Post-ethanol HICs were scored hourly from 2 to 2 h post-ethanol in R2 homozygotes (black squares), R2 heterozygotes (gray squares), and WT littermates (open squares) (*N* = 27, 44, and 16 mice per genotype, respectively). After 4–5 h, convulsion scores increased above baseline, indicating a state of withdrawal hyperexcitability that peaks about 6–8 h post-administration. Data represent the HIC score (mean ± SEM). **(C)** Alcohol withdrawal severity scores (corrected mean AUC ± SEM) for R2 homozygote, R2 heterozygote and WT littermates are shown. ^∗∗^Significantly different (*p* < 0.005) than WT littermates; ^#^Significantly different (*p* < 0.01) than R2 homozygotes.

The present studies seek to elucidate a mechanism(s) involved in the actions of a QTL with a large effect on genetic predisposition to alcohol withdrawal. We previously established the impact of a distal chromosome 1 QTL (*Alcw1/Alcdp1*) on genetic vulnerability to alcohol withdrawal convulsions in mice using a small donor segment congenic (R8; Kozell et al., 2008). Genotypic analysis determined the minimal introgressed interval of the newly created R2 congenic to be 10.2 Mb (164.3–174.5 Mb; maximal 164.1–174.6 Mb; build GRCm38), which encompasses the entire R8 1.1 Mb donor interval (**Figure 1A**). Following acute ethanol administration, HIC scores of R2 homozygotes, heterozygotes, and WT littermates increased above baseline, indicative of rebound hyperexcitability (a withdrawal sign), beginning between 4 and 6 h post-ethanol administration, and peaking in severity approximately 7–8 h post-ethanol (**Figure 1B**). Baseline HICs did not differ among R2 homozygotes, heterozygotes, and WT littermates (0.38 ± 0.15, 0.87 ± 0.17, and 0.55 ± 0.21, respectively, *F*_2,95_ = 2.2, *p* = 0.12, NS). However, alcohol withdrawal severity scores (**Figure 1C**) were significantly different among the three genotypes (*F*_2,94_ = 6.80, *p* = 0.002), with less severe withdrawal in R2 homozygotes than heterozygote and WT littermates (*p* = 0.007 and *p* = 0.004, respectively). Taken together with previous studies demonstrating that withdrawal following chronic and acute alcohol exposure is significantly more severe in R8 congenic compared to appropriate background strain animals (Kozell et al., 2008), these results are consistent with the conclusion that the influence of *Alcw1/Alcdp1* on alcohol withdrawal severity is significant on both high and low withdrawal genetic backgrounds.

### *Alcw1/Alcdp1* Affects Genome-Wide Differential Expression (DE)

**FIGURE 2:**
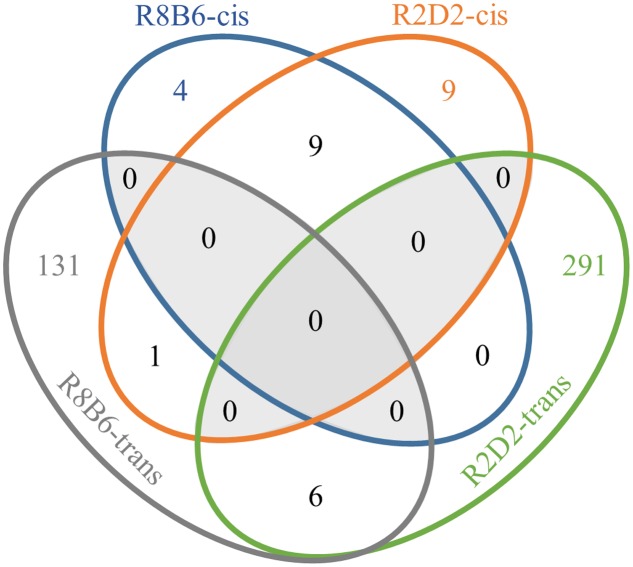
**Venn diagram illustrating correspondence of R8 vs. B6 differentially expressed genes and R2 vs. D2 differentially expressed genes**.

We next compared genome-wide gene expression in *Alcw1/Alcdp1* congenics (R2 and R8) to appropriate background strain animals (D2 and B6, respectively) using naïve animals to minimize the effects of individual differences in alcohol response. These data can thus not only inform genetic predisposition to withdrawal, but also potentially inform additional behaviors influenced by *Alcw1/Alcdp1* allelic status (pleiotropy). We used whole brain to assess gene expression in all critical brain regions, and we identified 147 significantly (*p* < 0.01) DE genes between R8 and B6: 13 (9%) are physically located within the R8 introgressed interval (*cis*-regulated), and the remaining 134 (91%) are located elsewhere in the genome (*trans*-regulated). As expected, with the relatively larger R2 donor interval (compared to R8 interval), more significantly (*p* < 0.01) DE genes were detected between R2 and D2 than between R8 and B6. Among the 316 DE genes, 19 (6%) are located within the R2 donor interval (*cis*-regulated), and 297 (94%) reside elsewhere (*trans*-regulated). All of the gene (microarray probe) comparisons, including the DE probes, are provided in **Supplementary Table 1**. Among the 147 DE genes identified in the R8 vs. B6 comparison, 16 were also identified in R2 vs. D2 (**Figure 2**). Nine of the shared DE genes are *cis-*regulated (*Adamts4, B4galt3, Copa, Fcgr3, Ncstn, Nit1, Ppox, Usp21*, and *Vangl2*), while six show *trans*-regulation in both (*Aaas, LOC100047619, Ccdc127, Gjc2, Lims2*, and *Pnkp).* The remaining shared DE gene (*Uck2*) is located within the R2 introgressed interval but beyond the R8 donor interval. As expected, DE is more conserved for *cis*-regulated genes than those showing *trans*-regulation.

For stringent DE validation by QPCR, we compared the *Alcw1/Alcdp1* congenic with the smaller introgressed interval (R8) to appropriate background strain (B6) animals (**Supplementary Table 2**). We tested 9 of the 13 genes implicated as DE by microarray and *cis*-regulated in an *Alcw1/Alcdp1* dependent manner. QPCR confirmed DE for 7 of these: *Ppox, Copa, Ncstn, Refbp2, Sdhc, Ufc1*, and *Ndufs2*. For the remaining two genes, QPCR detected either no evidence of DE (*Nit1*) or DE in the opposite direction of microarray (*Adamts4*). Differential results for microarray and QPCR data are not unusual (Morey et al., 2006), as both are based on probe hybridization which can detect transcript variation. Probe quality can also affect results, with QPCR being more sensitive than microarray. Furthermore, with markedly more alternative splicing events in brain than other tissues (Xu et al., 2002; de la Grange et al., 2010), the impact of splicing variation on probe specificity may contribute to the expression discordance we observed. Different sample sets were used for microarray and QPCR analyses, so batch effects may also contribute. We further tested by QPCR three genes within the R8 congenic interval that either lacked a microarray probe or had very low signal, and detected DE for all three: *Apoa2, Atf6*, and *Tstd1.* Three additional *cis-*regulated genes did not show DE on microarray, but were indicated as such by QPCR: *Nos1ap, Pex19*, and *Usf1*. We considered all of the *cis*-regulated genes demonstrating DE by QPCR to be candidate QTGs in subsequent *Alcw1/Alcdp1* analyses, which is further explored below.

To confirm *trans*-regulated genes, we prioritized genes recognized as potential interaction partners with R8 interval *cis*-regulated genes. QPCR confirmation for *trans*-regulated genes was not as high; only two of six genes implicated by microarray showed significant DE in the corresponding direction (*Mt2, Ndufa10*), while the other four showed DE in the opposite direction (*Eif2a, Cox6b2, Atp6ap1*, and *Atf4*). Six *trans*-regulated genes were not represented on the array or had sub-threshold signal, two of which (*Snrp1c, Wdfy1*) showed DE by QPCR. Importantly, out of 26 *trans*-regulated genes showing no DE on microarray, QPCR detected significant DE for 20 (*Taz, Nudt3, Ndufv1, Ndufb6, Mt1, Jam2, Itpka, Hspa5, Gclc, Gad1, Eif2a, Ddit3, Crh, Cox6a2, Cebpb, Bex1, Bdnf, Atp1b2, Asns*, and *Aldh2*).

We then explored potential functional relevance for genotype-dependent DE (*p* < 0.01) by assessing both congenic datasets for overlap using Molecular Signatures Database and Hallmark gene sets (see Materials and Methods). For these analyses we only used those on the microarray and not the additional data from QPCR because those were selected based on known interactions or pathways. The R8/B6 DE genes significantly (FDR < 0.05) overlapped with four Hallmark gene sets: Fatty_Acid_Metabolism, UV Response_Up, Myogenesis, and Oxidative_Phosphorylation. The R2/D2 DE genes significantly (FDR < 0.05) overlapped with 17 Hallmark gene sets, including E2F_Targets, G2M_Checkpoint, and Unfolded_Protein_Response (see **Supplementary Table 1** for full list of results). Only one set (UV Response_Up) had significant overlap in both R8/B6 and R2/D2 comparisons, but lacked any DE genes in common. Such non-overlap in Hallmark gene sets between reciprocal congenics could be due to the larger interval (and thus many more genes) in R2 compared to R8, genetic background effects (Liang et al., 2010), or the fewer number of individuals included in the R2/D2 dataset.

### WGCNA Identifies Consensus *Alcw1/Alcdp1* Networks in Reciprocal Congenics

Standard DE analysis can be limited to explain complex traits, which rely on the concerted effects of many genes (Gaiteri et al., 2014). To explore the more subtle organization and interactions affected by *Alcw1/Alcdp1*, we performed parallel WGCNA (Langfelder and Horvath, 2008) on both congenic/background genome-wide expression datasets. WGCNA uses unsupervised clustering to reduce large gene expression datasets into a network of modules defined by correlated expression patterns.

**FIGURE 3:**
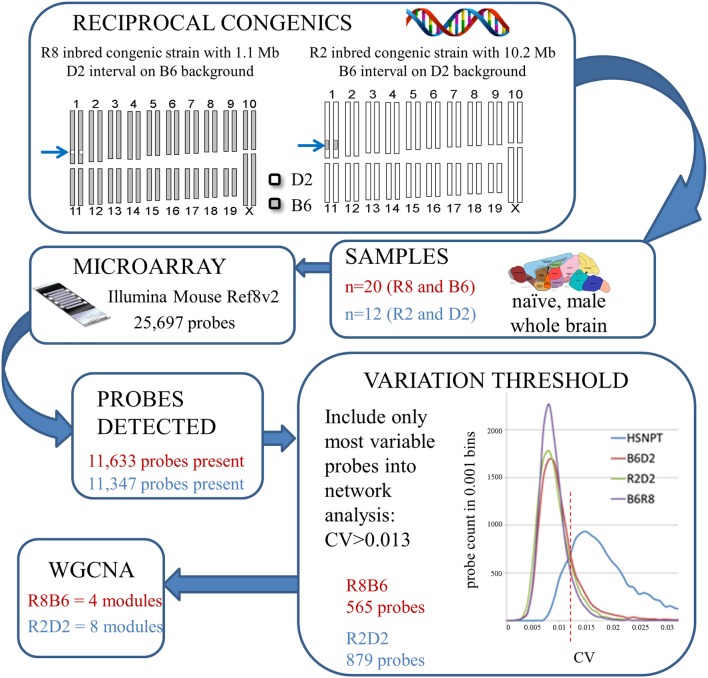
**Microarray and parallel weighted gene co-expression network analysis (WGCNA) workflow summary for each comparison of congenic and background strain**.

Two consensus co-expression networks were constructed, R8B6 and R2D2, consisting of 565 and 879 probes, respectively, that met inclusion criteria, including CV threshold. A schematic of the workflow is shown in **Figure 3**. Given the relatively small degree of genetic variation among *Alcw1/Alcdp1* congenic and background strain animals, network sizes are smaller than we have previously observed for WGCNA using heterogeneous stocks and other populations. Four modules in R8B6 and eight modules in R2D2 were defined by hierarchical clustering. These modules were assigned arbitrary colors, with total number of probes in each module given in parentheses: R8B6_blue_ (125), R8B6_brown_ (246), R8B6_green_ (140); R2D2_black_ (76), R2D2_brown_ (91), R2D2_green_ (157), R2D2_greenyellow_ (221), R2D2_pink_ (73), R2D2_purple_ (149), R2D2_turquoise_ (111). The gray module is reserved for genes not readily assigned to any other module (Langfelder and Horvath, 2007): R8B6_gray_ (54) and R2D2_gray_ (1). Probes and corresponding genes for each module are listed in **Supplementary Tables 3** and **4**. Potential module hubs are elaborated on in the discussion.

### Network Validation via Module Quality

Demonstrating statistical reproducibility of gene co-expression modules is critical for ensuring valid network architecture, i.e., ensuring that module clustering is not an artifact of batch effects. We used a bootstrapping procedure comparing average module connectivity to that of random gene groups to confirm network partition accuracy (Langfelder et al., 2011; Metten et al., 2014). This procedure generates *Z* scores for each module as a measure of quality: >2 is considered moderate and >10 is considered high. Two of the R8B6 modules are high quality (*Z*_brown_ = 11.5, *Z*_green_ = 20.5), one is moderate quality (*Z*_blue_ = 8.4), and as expected, the gray module does not vary from random connectivity (*Z*_gray_ = 1.4). Five of the R2D2 consensus modules are high quality (*Z*_green_ = 21.2, *Z*_greenyellow_ = 19.6, *Z*_pink_ = 17.8, *Z*_purple_ = 21.2, *Z*_turquoise_ = 10.3), and two are moderate quality (*Z*_black_ = 4.6, *Z*_brown_ = 8.0). A quality score could not be determined for R2D2_gray_ because it contains only one gene.

### An Oxidative Phosphorylation (OXPHOS) Module Is Common to Reciprocal Congenic Networks

As in DE analyses, we performed a preliminary query on the R8B6 and R2D2 WGCNA consensus networks for existing overlap in the Hallmark gene sets of the Molecular Signatures Database. Overlap of modules in both reciprocal congenic consensus networks with well-curated gene sets may indicate common neurobiological mechanisms contributing to the influence of *Alcw1/Alcdp1* on alcohol withdrawal severity. Such co-expression patterns are frequently subtle, involve different sets of genes, and are thus not detected at the relatively low level of resolution afforded by DE analysis alone (Gaiteri et al., 2014).

These analyses revealed remarkable correspondence between multiple modules in both co-expression networks and the Hallmark gene set for Oxidative_Phosphorylation (OXPHOS) (**Supplementary Table 3** for R8B6 and **Supplementary Table 4** for R2D2). Specifically, five high quality modules (R8B6_green_, R8B6_blue_, R8B6_brown_, R2D2_green_, and R2D2_greenyellow_) significantly overlapped with the OXPHOS Hallmark gene set (FDR *q* = 2.2 × 10^-5^, 1.4 × 10^-2^, 4.7 × 10^-2^, 6.9 × 10^-6^, 3.0 × 10^-6^, respectively), indicating the presence of functionally relevant co-expression in both reciprocal congenics despite the lack of detection by DE analysis alone.

### Module Commonality in Reciprocal Congenics

**FIGURE 4:**
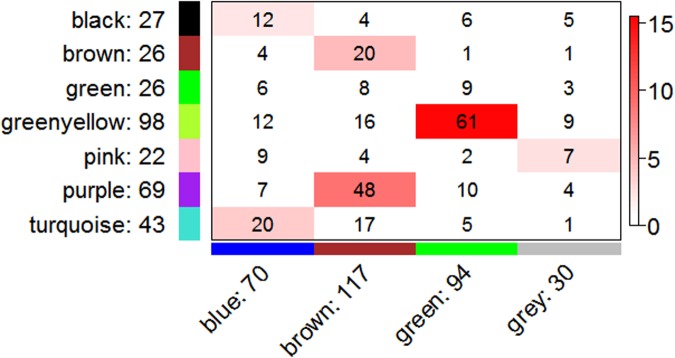
**Correspondence of co-expression in R8B6 and R2D2 consensus network modules.** Rows represent one R2D2 module (labeled by color and text), and columns represent one R8B6 module. Numbers in each cell indicate gene counts in the intersection of the corresponding modules. Coloring of the table encodes - log(p), with significance as Fisher’s exact test *p*-value for overlap of the two modules. Greater significance is indicated by a more intense red color.

To further resolve a potential unifying mechanism, we assessed shared membership between co-expression modules in the R8B6 and R2D2 consensus networks (**Figure 4**), which have 311 genes in common. The most significant overlap was observed between R8B6_green_ and R2D2_greenyellow_, with 61 genes in common. As shown above, these two modules also have the most significant overlap with the OXPHOS Hallmark gene set. Collectively, these results strongly support validity of the OXPHOS modules on both background strains and highlight the convergence of co-expression patterns that are not dependent upon background strain.

### Network Modules Most Correlated to Genotype

One overall goal of these studies is to understand the genetic basis of the significant, measurable variation in alcohol withdrawal severity observed behaviorally in animal models. To this end, we analyzed four strains with such demonstrable behavioral variation (two *Alcw1/Alcdp1* congenic models and appropriate background strain animals) for genetic factors that may putatively contribute. These genetic factors can thus be considered a phenotype of alcohol withdrawal severity and a molecular biomarker of predisposition (vulnerability). Importantly, to focus on the latter, as well as avoid potential confounds of alcohol on gene expression, here we used naïve animals in all studies. In network analyses, modules correlated to genotype are associated with the trait of alcohol withdrawal vulnerability. Each module was correlated to the genotype using standard WGCNA methods for gene significance (Langfelder and Horvath, 2008). Three modules showed significant correlation (R8B6_gray_ *r* = 0.9, *p* = 5.8 × 10^-7^; R2D2_pink_ *r* = -0.9, *p* = 2.6 × 10^-4^; R2D2_brown_ *r* = -0.7, *p* = 1.2 × 10^-2^). Two of these modules contain the highest percentage of genes located within their respective congenic intervals (R8B6_gray_ = 8, 15%; R2D2_pink_ = 16, 22%). R2D2_pink_ also contains 26 genes located beyond the congenic interval that are significantly correlated to genotype. In contrast, all 38 genes correlated to genotype in the R2D2_brown_ module are located beyond the congenic interval (i.e., *trans*-regulated genes).

### Network Modules Correlated to Cell Type

Using specific cell type data (Cahoy et al., 2008) based on our previous methods (Metten et al., 2014), we tested modules for enrichment in neuronal cell types, oligodendrocytes, and astrocytes. No significant cell type enrichment was found for any of the modules in either dataset.

### OXPHOS Modules Are Common to Diverse Alcohol-Related Networks

We identified a common genetic signature (co-expression modules) in chromosome 1 reciprocal congenics; however, this does not necessarily conclude specific influence of the chromosome 1 interval and its accompanying alcohol withdrawal severity locus. It is possible that the co-expression modules we detected are actually a resting state pattern common to many populations. Remarkably, we and others have observed OXPHOS modules in WGCNA of multiple AUD datasets in both animal models and human studies of alcohol dependence, thereby validating biological relevance of these pathways (Gaiteri et al., 2014). To minimize additional complexity contained in R2 data due to the substantially larger introgressed region than R8, we limited comparisons with other datasets to the most robust OXPHOS R8B6 module (R8B6_green_). Using Gene Weaver (Baker et al., 2012), we identified two modules in other previously published datasets that display significant gene membership correspondence with the OXPHOS R8B6_green_ module. First, network analyses in B6D2-derived mice selected for dual traits of high alcohol consumption and low alcohol withdrawal severity and *vice versa* (SOT and NOT lines, respectively; Metten et al., 2014) identified a co-expression module (SOT/NOT_lightpink4_) with pronounced corKME disruption that overlapped with the OXPHOS Hallmark gene set (FDR *q* = 7.8 × 10^-20^). SOT/NOT_lightpink4_ was also the module most enriched for DE genes between the SOT and NOT lines (*p* < 2 × 10^-88^). Sixty-six genes within this module are shared in the R8B6_green_ module, including *Sdhc*, an *Alcw1/Alcdp1* candidate QTG with multiple lines of supporting evidence (further explored below). Given that dual selection included the alcohol withdrawal severity behavioral trait, this convergence indicates OXPHOS could be an important contributory genetic mechanism.

Gene Weaver also identified fifty-seven genes from the R8B6_green_ OXPHOS module in common with the blue module from network analyses on mice selected for haloperidol-induced catalepsy (Iancu et al., 2012). These strains were derived from the heterogeneous stock of a 4-way cross of B6, D2, BALB/cJ, and LP/J strains (HS4). The HS4_blue_ module also significantly overlaps the OXPHOS Hallmark gene set (FDR *q* = 2.9 × 10^-11^). Furthermore, considering that a haloperidol response QTL is centered less than 5 Mb distal to the R8 congenic interval on chromosome 1, it is feasible that the OXPHOS co-expression module was selected in the haloperidol response by proximity to the alcohol withdrawal QTL. While confirmation is necessary, these results support involvement of this distal chromosome 1 region in an OXPHOS mechanism correlated to behavior.

Our results pointing to the involvement of an OXPHOS network in risk for alcohol withdrawal in naïve animals lead to the question of whether this co-expression pattern may be affected by alcohol exposure. In a literature survey, we identified several additional reports of significant OXPHOS module contribution to co-expression networks detected in alcohol-exposed mouse brain. Among alcohol-affected modules in B6 amygdala synaptoneurosomes (Most et al., 2015), three significantly overlapped the OXPHOS Hallmark gene set (FDR *q* < 5.6 × 10^-13^). Using DE and co-expression analyses, Nunez et al. (2013) identified miRNA-mRNA interaction networks responding to ethanol consumption in mice, and among five alcohol-responsive modules, the yellow module was enriched in DE genes, correlated to alcohol consumption, and significantly overlapped the OXPHOS Hallmark gene set (FDR *q* = 2.3 × 10^-20^).

Importantly, OXPHOS modules were also seen in two systems level co-expression studies of human postmortem brain. Ponomarev et al. (2012) compared epigenetic co-expression in superior frontal cortex and amygdala (basolateral and central nucleus) of alcoholics to control subjects. Alcohol abuse was associated with global gene expression changes in all three brain regions, co-expression patterns were highly conserved, and all three regions contained modules that overlap with OXPHOS Hallmark gene set (all FDR *q* < 1.3 × 10^-6^). Both basolateral amygdala and superior frontal cortex were alcohol-responsive, the latter with high OXPHOS correspondence (FDR *q* = 9.3 × 10^-35^). Zhang et al. (2014) identified four co-expression modules associated with AUD in postmortem dorsolateral prefrontal cortex, one of which significantly overlapped with OXPHOS Hallmark gene set (FDR *q* = 7.4 × 10^-19^). While the authors’ conclusion that “expression alterations in this group of genes could either make subjects more vulnerable to AUDs, or reflect the results of that vulnerability,” our results with naïve animals lend strong support to the idea that preexisting vulnerability emerges, at least in part, from a mechanism involving OXPHOS.

### Antioxidant Pretreatment Significantly Mitigates Alcohol Withdrawal in Mice

**FIGURE 5:**
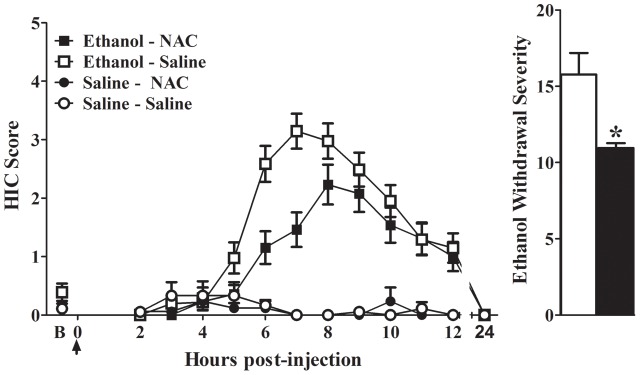
**(A)** Acute alcohol withdrawal was indexed by HICs in ethanol- or saline-exposed groups pretreated with NAC or vehicle (saline). Mice were pretreated (-48 and -24 h) with NAC or vehicle and scored twice for baseline HICs (indicated by B) immediately before administration of 4 g/kg ethanol or saline (arrow = injection at time 0), and hourly 2–12 h post-injection. After 4–5 h, convulsion scores increased above baseline, indicating a state of withdrawal hyperexcitability that peaks about 6–8 h post-administration. HIC scores return to baseline within 24 h. **(B)** Alcohol withdrawal severity (corrected AUC, mean ± SEM) for NAC and vehicle-pretreated ethanol groups. For ethanol-exposed animals, NAC-pretreatment significant decreased withdrawal severity compared to vehicle (^∗^*p* < 0.0005). Data represent the strain mean ± SEM (*n* = 38, 41, 17, and 18 mice per group, respectively).

To validate the contribution of an OXPHOS co-expression mechanism to alcohol withdrawal vulnerability, we sought a pharmacological agent that could manipulate the behavioral phenotype. NAC is well-known to broadly affect oxidative homeostasis and is FDA-approved for clinical treatment of acetaminophen toxicity, bronchitis, and chronic obstructive pulmonary disease. Due to a robust alcohol withdrawal phenotype, we assessed D2 strain mice for NAC effects on withdrawal severity. Naïve mice were pretreated with NAC (300 mg/kg, i.p.) or vehicle (saline) at 48 and 24 h prior to ethanol (4 g/kg, 20% v/v in saline, i.p.) or saline (control) administration. Data were collapsed across sex as there was no effect of sex (*F*_1,103_ = 0.01, *p* = 0.95), or any interactions between sex and pretreatment (NAC or saline; *F*_1,103_ = 0.03, *p* = 0.85), sex and treatment (ethanol or saline; *F*_1,103_ = 0.08, *p* = 0.78), or sex × pretreatment × treatment (*F*_1,103_ = 0.4, *p* = 0.52). No differences were observed in baseline (pre-ethanol or pre-saline control) HIC scores among the four groups (ethanol – NAC 0.1 ± 0.1, saline – NAC 0.1 ± 0.1, ethanol – saline 0.1 ± 0.1, saline – saline 0.2 ± 0.1; *F*_1,109_ = 0.04, *p* = 0.6). HIC scores increased above baseline at ∼4–5 h post-injection and peaked at ∼6-8 h in both ethanol-treated groups (**Figure 5**). Significant differences were seen for NAC compared to vehicle pretreatment (*F*_1,107_ = 6.3, *p* = 0.014), ethanol or saline treatment (*F*_1,107_ = 85.8, *p* = 2.3 × 10^-11^), and a pretreatment × treatment interaction (*F*_1,107_ = 4.9, *p* = 0.030). Notably, *post hoc* Tukey analysis showed significantly less severe withdrawal in NAC-pretreated compared to vehicle-pretreated mice during ethanol withdrawal (*p* = 3.3 × 10^-4^; **Figure 5**). No differences were detected between the NAC and vehicle pretreated groups (0.7 ± 0.4 and 1.1 ± 0.4, respectively; Tukey *p* = 0.99).

## Discussion

### *Alcw1/Alcdp1*, OXPHOS, and NAC

Previous WGCNA analyses have detected relevant gene expression changes in genetic models with limited diversity, such as knockouts (MacLennan et al., 2009; Menezes et al., 2012; Deshpande et al., 2016; Provenzano et al., 2016; Stacey et al., 2016). Here, applying WGCNA to reciprocal congenic and background strains revealed the preservation of gene expression networks across genetic backgrounds, further illustrating the value of using systems approaches in robust animal models of human disease, even when genetic diversity is minimal. Our identification of a co-expression module (OXPHOS) common to two congenic models highlights the power of WGCNA to uncover meaningful, strain-dependent variation in gene expression, despite a lack of such detection by traditional DE analyses. Considering the well-known impact of background strain on DE, the use of reciprocal background congenics can offer valuable confirmation of pathways or mechanisms central to the phenotype. The presence of an OXPHOS co-expression module in *both* R8B6 and R2D2 networks provides strong evidence that a gene(s) in the shared congenic region (corresponding to the 1.1 Mb *Alcw1/Alcdp1* interval) plays a key role in pathways related to oxidative homeostasis.

In addition, significant correspondence of R8B6_green_ OXPHOS module with several co-expression modules from other datasets related to alcohol withdrawal or alcohol affected co-expression modules in human and animal studies supports the interpretation that the R8B6_green_ OXPHOS module is a valid, robust co-expression module. Furthermore, detecting *only* these in common also implies that OXPHOS enrichment is not a universal regulatory mechanism found in many populations (Langfelder et al., 2012); if this were the case, we would expect to detect this co-expression module across many naïve mouse models.

Our previous detailed molecular analyses of the *Alcw1/Alcdp1* interval revealed remarkable genetic variation and prioritized a list of QTG candidates that, while functionally diverse, are notably over-represented by genes in OXPHOS pathways (Denmark and Buck, 2008). Importantly, the evidence we provide here that a brain antioxidant (NAC) can mitigate the behavioral expression of severe alcohol withdrawal in mice implicates a contribution of OXPHOS-related mechanism(s) to the withdrawal phenotype.

### Candidate QTGs and Co-expression Network Hubs

The discovery of *Alcw1/Alcdp1* led to genome-wide analyses for genetic determinants and/or potential biomarkers of alcohol withdrawal vulnerability, including potential QTGs that may drive genotype-dependent DE patterns underlying behavioral variation in alcohol withdrawal severity. Systems approaches such as the WGCNA used here are unbiased strategies to interrogate the *Alcw1/Alcdp1-*driven OXPHOS co-expression network for highly connected genes. The highly related measures of module membership (kME) and intramodular connectivity (kWithin) describe the degree of relationship of a gene to a ME and the degree of connectivity between a gene and other genes in the module (Horvath and Dong, 2008; Oldham et al., 2008) and can be reliably used to designate hub rank (**Supplementary Tables 3** and **4**). The most highly connected genes (hubs) in a module are biologically important in both lower organisms as well as mammals (Edwards and Palsson, 2000; Dipple et al., 2001; Jeong et al., 2001). Such members may represent high priority therapeutic candidates, since disruption of hubs often leads to functional impairment of the entire network (Jeong et al., 2001). However, disruption of a hub gene may not be therapeutically cogent due to far-reaching effects beyond the phenotype of interest. Here we discuss evidence for the most promising candidate QTGs and hubs, particularly addressing evidence for AUD interactions and involvement in OXPHOS mechanisms.

*Ndufs2* encodes a core protein [NADH dehydrogenase (ubiquinone) Fe-S protein 2] crucial to mitochondrial respiration and is a promising *Alcw1/Alcdp1* QTG candidate. *Ndufs2* is *cis*-regulated and demonstrates significant DE between R8 congenic and background strain animals. *Ndufs2* also contains a coding region SNP between the B6 and D2 progenitor strains with predicted functional relevance (Denmark and Buck, 2008). Mutation of the *Caenorhabditis elegans* ortholog causes redox stress and ethanol hypersensitivity (Kayser et al., 2003). *Ndufs2* mRNA content is regulated by ethanol in the amygdala (Most et al., 2015).

*Sdhc* (Succinate dehydrogenase complex, subunit C) is another *Alcw1/Alcdp1* QTG candidate significantly DE between *Alcw1/Alcdp1* congenic and WT mice and contains three functionally critical B6/D2 coding region SNPs (Denmark and Buck, 2008). SDHC is an integral membrane protein required for membrane-anchoring and functional assembly of respiratory Complex II and a convergence point where substrate metabolism is coupled to ATP-generating OXPHOS. Expression is ethanol-regulated in the amygdala (Most et al., 2015). *Sdhc* is also contained in the significant OXPHOS (SOT/NOT_lightpink4_) module of the SOT/NOT selected lines for dual traits of alcohol consumption and withdrawal (Metten et al., 2014).

Activating transcription factor 6 (*Atf6*) is an *Alcw1/Alcdp1* QTG candidate encoding a transmembrane endoplasmic reticulum (ER) protein cleaved in response to ER stress. While there are no B6/D2 SNPs, *Atf6* is DE between *Alcw1/Alcdp1* congenic and background strain mice and is a candidate QTG in reciprocal QTLs for ethanol withdrawal and drinking in a heterogenous stock (HS4) population (Hitzemann et al., 2009).

With high kME and kWithin values, *Hsp90aa1* (Heat shock protein 90, alpha, class A member 1) is a hub candidate in the reciprocal congenic OXPHOS modules. *Hsp90aa1* is also co-expressed with *Ndufs2* in an alcohol-regulated module via miRNA interactions in mouse frontal cortex (Nunez et al., 2013). HSP90AA1 is decreased in B6 mouse cortex in a chronic intermittent ethanol exposure paradigm (Gorini et al., 2013) and is decreased in macrophages after short-term, and increased after long-term, ethanol exposure (Mandrekar et al., 2008). Hydrogen peroxide-induced renal cell death partly degrades HSP90AA1 and is associated with increases in lipid peroxidation, both of which can be prevented by NAC (Nowzari et al., 2000).

*Map2k1* (Mitogen-activated protein kinase 1) is a hub candidate in our studies that is also implicated in multiple alcohol studies, including WGCNA in SOT/NOT selected lines where *Map2k1* is a hub in the OXPHOS (SOTNOT_lightpink4_) module enriched for MAP kinase (MAPK) signaling-associated transcripts (Metten et al., 2014). Expression profiling also identifies changes associated with ethanol withdrawal in the MAPK pathway in B6 and D2 mice (Daniels and Buck, 2002). OXPHOS-generated ROS activate MAPK pathways (McCubrey et al., 2006; Son et al., 2013) and antioxidants block MAPK activation (Son et al., 2013).

Heat shock protein 5 *(Hspa5)* is a moderate OXPHOS hub candidate that has many other factors to support it as a candidate: it resides in a SOT/NOT QTL, is DE between the selected lines (Metten et al., 2014), and is implicated in alcohol consumption (Tarantino et al., 1998; Belknap and Atkins, 2001; Bell et al., 2009). Although there are no B6/D2 SNPs, QPCR (**Supplementary Table 2**) confirms *trans*-regulation of *Hspa5* by the *Alcw1/Alcdp1* interval, as well as *trans*-regulation of two interaction partners (*Atf6, Atf4*). *Atf4* is a transcription factor that activates the *Hspa5* promoter (Luo et al., 2003). *Hspa5* is a member of the MAPK signaling pathway (hub candidate listed above, Chen et al., 2000) and is a biomarker of ER stress (Hampton, 2000; Harding et al., 2002). HSPA5 (also called GRP78/BIP), is an ER chaperone protein which retains ATF6 in the ER by inhibiting its Golgi localization signals. Dissociation of HSPA5 during ER stress allows ATF6 to be transported to the Golgi (Shen et al., 2002). Chronic exposure to moderate levels of ethanol in neurons (*in vitro*) increases the levels of HSPA5 (Romero et al., 2015). *Hspa5* expression is also altered by chronic ethanol consumption in rats (Bell et al., 2009).

Each of these candidate QTGs or hubs requires follow up studies to confirm them as a biomarker or as a potential target for therapeutic intervention. Current molecular techniques offer many options to validate of these candidates. For example, traditional knockout animals could confirm gene effect on alcohol withdrawal, and newer mutation techniques such as CRISPR/Cas9 could validate genetic effects on withdrawal severity. Next we discuss the OXPHOS mechanism in a network of co-expressed genes and the role of NAC as a pharmacological agent to disrupt the OXPHOS network.

### Oxidative Stress and the Brain

The mitochondrial respiratory chain (MRC) consists of four protein complexes that use electron transport to drive OXPHOS and generate ATP, producing ROS as byproducts that are normally balanced by multiple antioxidant mechanisms. The brain is particularly vulnerable to redox imbalance and ROS accumulation due to (1) high oxygen metabolism, (2) relative under-abundance of antioxidant defenses, and (3) high polyunsaturated fatty acid content of neural membranes, which are ROS substrates (Sun and Sun, 2001). A growing body of evidence has uncovered natural variation in redox homeostasis among B6, D2, and B6D2-derived mice, which disproportionately affects tissues with high energy demand, including brain (Bhave et al., 2006; Misra et al., 2007). We recently demonstrated that B6 and D2 exhibit remarkable differences in brain MRC organization and function (Buck et al., 2014). Alcohol interferes with brain oxidative homeostasis, as metabolic byproducts of alcohol (including acetaldehyde and ROS) drive OXPHOS, impair antioxidant defenses, and can persist long after the initial exposure (Sun and Sun, 2001; Bailey, 2003). In contrast, barbiturate exposure appears to have neutral or anti-oxidative effects (Smith et al., 1980; Almaas et al., 2000; Ueda et al., 2007). While alcohol-induced oxidative damage is well-established, changes during alcohol withdrawal remain mostly uninvestigated. The few available rodent studies show increased brain ROS several hours after ethanol exposure (Dahchour et al., 2005) and a correlation with withdrawal seizure severity (Vallett et al., 1997).

### *N*-Acetylcysteine (NAC)

The Comparative Toxicogenomics Database (CTD; Davis et al., 2015) provides manually curated data about the human health consequences of chemicals and represents a triad of chemical-gene, chemical-disease, and gene-disease interactions. According to CTD, NAC interacts with 835 genes, including R8B6_green_ module member human homologs: *ACTA2, CHKA, HSPA5, ID2, JAK1, MAP2K1, PCNA*, and *YWHAZ*. Of these 835 genes, 48 are annotated as “response to oxidative stress” (GO:0006979, *p* = 5.8 × 10^-23^). Another database, Drug2gene (Roider et al., 2014), shows evidence of 387 human NAC-affected genes, including R8B6_green_ module homologs: *HSPA5, JAK1, PCNA, TFRC*, and *CALR*. Of these 387 genes, 38 are annotated as “response to oxidative stress” (GO:0006979, *p* = 1.0 × 10^-23^). According to Drug2gene, CTD or both, NAC also interacts with *ATF6*, a *cis*-regulated gene in the R8 interval and several confirmed R8 *trans*-regulated genes (**Supplementary Table 2**): *ALDH2*, *EIF2A, DDIT3*, *GCLC*, *ATF3, ATF4*, *HMOX1*, *CEBPB*, *BDNF, MT1*, *MT2*, and *NDUFB6.*

Among others, NAC is a mitochondria-targeted antioxidant with powerful radical scavenging activity (Samuni et al., 2013) with an established safety record in adults and children and approved by the FDA since 1963 (McClure et al., 2014). NAC administration enhances the redox potential of glutathione (GSH)/glutathione disulfide (GSSG), the cysteine/cystine cycle, and cystine/glutamate antiporter activity, with a net effect of lessening oxidative cellular dysfunction (Berk et al., 2013). Ethanol induces oxidative stress in many tissues and contributes significantly to the mechanisms by which ethanol produces liver injury (Cederbaum et al., 2009). Antioxidants appear hepatoprotective in some models (Wang et al., 2015; Xiao et al., 2015), including inhibition of alcohol-induced oxidative stress by NAC co-administration in rat liver (Ozaras et al., 2003). In another study of alcohol-exposed rat liver, NAC inhibited mitochondrial biogenesis genes, but did not prevent mitochondrial damage (Caro et al., 2014). To our knowledge, only one study has assessed potential NAC effects after alcohol cessation. Schneider et al. (2015) reported that 4 days of NAC treatment prevented alcohol cessation related decreases in open field activity and prevented increases in peripheral blood corticosterone and leptin levels otherwise observed in rats after 5 days of alcohol cessation (Schneider et al., 2015). However, there are numerous differences between this study and the current one, including genetic model, alcohol exposure model, assessment of peripheral vs. centrally mediated effects of NAC, and the dose NAC given.

In humans, NAC oral bioavailability is 4–10% and may be limited by nausea or vomiting (Borgström et al., 1988) in humans. Published reports about whether NAC delivered orally crosses the blood brain barrier (BBB) are contradictory (see Samuni et al., 2013 for review). With such low bioavailability, therapeutic effects of oral NAC are likely secondary (e.g., induction of glutathione synthesis) and could also be true of intravenous administration (Fishbane et al., 2004). Due to restricted BBB passage of NAC, the amide form (NACA) may be more therapeutically relevant. NACA is significantly more lipophilic, allowing ready BBB and cell penetration and subsequent scavenging of free radicals (Semple, 2014). Future studies of the potential of NAC to mitigate withdrawal severity may thus benefit from testing this amide form.

### Limitations

While a primary goal of these studies was to determine genetic influences on gene expression, epigenetic influences are also likely at play. These analyses may detect the effects of those influences, but miss the specific cause. Additionally, resolving DE and WGCNA in specific brain regions involved in alcohol withdrawal circuitry (e.g., substantia nigra pars reticulata or prefrontal cortex) may reveal more detailed co-expression networks and/or DE genes by reducing potential noise vs. the whole brain assessment performed here. The use of whole brain may also explain our lack of detection of any cell-type enrichment. Since we evaluated only males, it remains to be determined to what extent our findings may generalize to females. With full containment of the distal chromosome 1 interval in both congenics, it was not possible to confirm its influence here. The small sample size also precludes us from determining the influence of additional factors such as cage or family effect. A final limitation is that we tested only one genotype for the NAC effects. Future directions will include analyses of the NAC effects on other genotypes, as well as gene expression analyses following alcohol and/or NAC treatments.

## Conclusion

Network-based approaches to human disease have multiple potential applications, even beyond identification of disease genes. These include better targets for drug development, more accurate biomarkers, and improved disease classification (Barabasi et al., 2011). Understanding the larger biological systems (of which genes are just one part) is critical to realizing the ultimate goals of personalized and precision medicine.

Taken altogether, our data implicate oxidative homeostasis/stress and *Alcw1/Alcdp1* as having key roles in genetic vulnerability for alcohol physical dependence and associated withdrawal and suggest OXPHOS as a potential pathway target for clinical management of alcohol withdrawal. Importantly, this leads to the hypothesis that antioxidants (e.g., NAC) may be appropriate therapeutic agents for reducing severity of alcohol withdrawal symptoms. Future studies will be needed in order to assess how OXPHOS co-expression modules change after alcohol exposure and withdrawal, to assess whether hub gene disruption affects behavioral expression of withdrawal, and to determine if other antioxidant pharmacotherapies (e.g., NACA) can also ameliorate withdrawal.

## Data

Microarray dataset is publically available via GEO at NCBI. Accession# GSE89281.

## Author Contributions

KB, NW, LK, and DD participated in the design of these studies. NW and DD performed the QPCR analyses. LK performed the behavioral experiments and analysis. NW performed the gene expression and network analyses. DD, NW, LK, and KB contributed to writing the manuscript. All authors have read and approve the final version of this manuscript.

## Conflict of Interest Statement

The authors declare that the research was conducted in the absence of any commercial or financial relationships that could be construed as a potential conflict of interest.

## Footnotes

1 www.r-project.org

2 www.bioconductor.org

## Supplementary Material

**TABLE 1 | Differential expression between R8 and B6 and overlap of significantly (*p* < 0.01) differentially expressed genes with MSigDB Hallmark gene sets.** Differential expression between R2 and D2 and overlap of significantly (*p* < 0.01) differentially expressed genes with MSigDB Hallmark gene sets.

**TABLE 2 | QPCR confirmation of differential expression of R8 *cis-* and *trans-*regulated genes.**

**TABLE 3 | R8B6 co-expression network analyses, with module assignment, kWithin (intramodular connectivity), kME (module membership), and GSP (gene significance *p* value).** Overlap of module genes with MSigDB Hallmark gene sets.

**TABLE 4 | R2D2 co-expression network analyses, with module assignment, kWithin (intramodular connectivity), kME (module membership), and GSP (gene significance *p* value).** Overlap of module genes with MSigDB Hallmark gene sets.
